# Impact of Pharmacist-Led Diabetes Self-Care Education on Patients With Type 2 Diabetes in Pakistan: A Randomized Controlled Trial

**DOI:** 10.3389/fphar.2022.754999

**Published:** 2022-02-09

**Authors:** Allah Bukhsh, Tahir Mehmood Khan, Pochamana Phisalprapa, Acharaporn Duangjai, Surasak Saokaew, Muhammad Sarfraz Nawaz, Hafiz Sajjad Ahmed, Bey-Hing Goh

**Affiliations:** ^1^ School of Pharmacy, Monash University, Jalan Lagoon Selatan, Malaysia; ^2^ Institute of Pharmaceutical Sciences, University of Veterinary and Animal Sciences, Lahore, Pakistan; ^3^ Division of Ambulatory Medicine, Department of Medicine, Faculty of Medicine Siriraj Hospital, Mahidol University, Bangkok, Thailand; ^4^ Unit of Excellence in Research and Product Development of Coffee, Division of Physiology, School of Medical Sciences, University of Phayao, Phayao, Thailand; ^5^ Center of Health Outcomes Research and Therapeutic Safety (Cohorts), School of Pharmaceutical Sciences, University of Phayao, Phayao, Thailand; ^6^ Unit of Excellence on Clinical Outcomes Research and Integration (UNICORN), School of Pharmaceutical Sciences, University of Phayao, Phayao, Thailand; ^7^ Unit of Excellence on Herbal Medicine, School of Pharmaceutical Sciences, University of Phayao, Phayao, Thailand; ^8^ Division of Social and Administrative Pharmacy, Department of Pharmaceutical Care, School of Pharmaceutical Sciences, University of Phayao, Phayao, Thailand; ^9^ Department of Pharmacy, Quaid-i-Azam University Islamabad, Islamabad, Pakistan; ^10^ Capital Hospital, Islamabad, Pakistan; ^11^ Biofunctional Molecule Exploratory (BMEX) Research Group, School of Pharmacy, Monash University Malaysia, Subang Jaya, Malaysia; ^12^ College of Pharmaceutical Sciences, Zhejiang University, Hangzhou, China

**Keywords:** type 2 diabees (T2D), self-care, diabetes knowledge, Hba1 C, pharmacist

## Abstract

**Introduction:** There is a little evidence on efficacy of pharmacy-based interventions on clinical outcomes of type 2 diabetes mellitus (T2DM) patients in Pakistan.

**Objective:** To appraise the impact of pharmacist-led self-care education on glycemic control, self-care practices and disease knowledge of T2DM patients with poor glycemic control (HbA1c ≥ 7%).

**Methods:** In this 6-months, randomized controlled trial (RCT), *n* = 75, T2DM patients seeking care at a diabetes clinic were randomized in to two groups. Intervention group (*n* = 38) received two face-to-face educational sessions (at enrollment and on week 12), whereas control group (*n* = 37) received usual care. Outcome measures such as glycemic control, self-care practices and disease knowledge were assessed at the time of enrollment and after 6-months in both groups.

**Results:** Thirty-three intervention and thirty-three participants from the control group completed the study. Mean glycated hemoglobin (% HbA1c) significantly reduced in the intervention group from 9.00 ± 1.43 to 8.09 ± 1.16 (*p* < .01). However, no significant change was observed in the control group (9.20 ± 1.24 to 8.93 ± .97; *p* = .06). Cohen’s d effect size of the intervention on HbA1c was .78. Percentage of participants achieving glycemic control (HbA1c < 7%) were significantly higher (*p* < .05) in the intervention group as compared to the control group (twenty-four vs. six), after 6 months of the trial. A significant (*p* < .01) improvement in mean scores for disease knowledge and self-care activities was also observed in the intervention group participants, whereas no significant improvements (*p* > .05) were observed in the control group.

**Conclusion:** The study demonstrated an improvement in glycemic control, disease knowledge and self-care activities of T2DM patients who received pharmacist-led educational intervention. The study findings support clinical significance of integrating pharmacy-based interventions in diabetes management.

## Introduction

Diabetes is one of the leading chronic diseases with a significant economic and public health burden worldwide. With 7.5 million people with diabetes, Pakistan has been ranked 10th for diabetes burden in the world (International Diabetes Federation, 2017). Currently, there are 4.6 million people with undiagnosed diabetes in Pakistan. If this scenario continues, the number of people with diabetes in Pakistan will be more than double (16.1 million) in 2045 (International Diabetes Federation, 2017).

Management of diabetes is challenging, as the patients have to adhere to many lifestyle modifications regularly. Such modifications include healthy eating habits, regular exercise, self-blood glucose testing, and adherence to medication (Inzucchi et al., 2012). Inadequate self-care practices have been reported in people with diabetes living in low- or middle-income countries (Sarkar et al., 2006; Jarab et al., 2012; Wishah et al., 2015). Recently published data show poor self-care activities among Pakistani people with diabetes (Bukhsh et al., 2018a; Bukhsh et al., 2019).

A comprehensive and culturally-sensitive intervention involving a multidisciplinary team approach is need of the hour to address this massively growing issue. Poor glycemic control and associated complications is one of the challenges being faced by low- and middle-income countries. In the International Diabetes Federations’ (IDF) Middle East and North Africa (MENA) region, Pakistan has the lowest cost (per person) for diabetes management (International Diabetes Federation, 2017).

Besides pharmaceutical care planning, pharmacists are playing a key role in managing chronic diseases by providing educational interventions. Studies have proven the beneficial value of pharmacists in improving self-care, disease knowledge and glycemic control among people with diabetes (Van Eikenhorst et al., 2017; Yaghoubi et al., 2017; Bukhsh et al., 2018b). A network meta-analysis of forty-three randomized control trials demonstrated that pharmacist-based interventions could significantly reduce the levels of glycated hemoglobin in the intervention group (Bukhsh et al., 2018c). Most of the studies included in this network meta-analysis were conducted in high-income countries (Bukhsh et al., 2018c).

To date, studies examining the impact of pharmacist-led educational intervention in achieving desired clinical outcomes are scarce in Pakistan. To address this knowledge gap, in the current study, we conducted a randomized control trial to examine the efficacy of a 6-month pharmacist-led diabetes educational intervention on type 2 diabetes patients with poor glycemic control, in one of the diabetes care clinics of Pakistan.

## Methodology

### Study Design and Setting

This 6-month, open-labelled, prospective, parallel group, randomized controlled trial was conducted from December 2017 to October 2018. The study was conducted at diabetes clinic of Capital Hospital, located in the capital city of Pakistan (Islamabad). The average diabetes patients’ turn over in this healthcare facility is about 80 per week. Ethics approval for the study was obtained from Monash University Human Research Ethics Committee (Ethics approval number 10817; approval date: 22nd September 2017). The protocol of this trial met Consolidated Standards of Reporting Trials (CONSORT) guidelines and American Medical Association (ADA) guidelines for medical care of people with diabetes (Association AD, 2002). This trial has been registered with Australian New Zealand Clinical Trials Registry (Registration No. ACTRN12617001327370; Registration date: 15th September 2017).

The details about demographic characteristics, self-care behaviors and disease knowledge of intervention and control group participants were collected at the start (baseline) and at the end (24th week) of the trial.

### Participants

The study sample included both male and female patients older than 30 years of age with a confirmed diagnosis of type 2 diabetes and a history of HbA1c ≥ 7% within the preceding month. Patients were excluded if they were involved in any educational trial related to diabetes in past 3 months, suffering with type 1 diabetes, gestational diabetes, cognitive impairment, and terminal illness.

An informed written consent for participation in the study was taken from the participants, after explaining them the study objective and procedure. Participants were randomized into intervention and control groups by using a computer-generated randomization list and allocation was concealed by using envelop method. The participants of intervention group and control group were called on alternate days to receive pharmacist-led educational intervention and usual care, respectively, so as to minimize the interaction among them and possible contamination. The sample size of the study was calculated on the basis of its ability to detect an effect size of 1% with standard deviation of ±1.4% (Sarkadi and Rosenqvist, 2004; Hayward et al., 2005; Ali et al., 2012; Chen et al., 2012; Moreira et al., 2015), 80% sampling power, and .05 significance level, at 6-month in the intervention group compared to the control group. A sample size of sixty-two was calculated (*n* = 31 each in the intervention and control group), but seventy five patients were recruited in the study, in order to compensate 20% attrition rate (Chow et al., 2007; Hulley et al., 2013).

One hundred and thirty eight potentially eligible patients were identified from the hospital records. However, *n* = 11 patients had HbA1c levels less than 7%, *n* = 48 had no recent HbA1c levels, and, *n* = 4 refused to participate in the trial due to hectic nature of their job or they were residing far away from the city. A total of seventy-five T2DM patients were recruited from those seeking medical services for their diabetes at Capital Hospital Islamabad.

**FIGURE 1:**
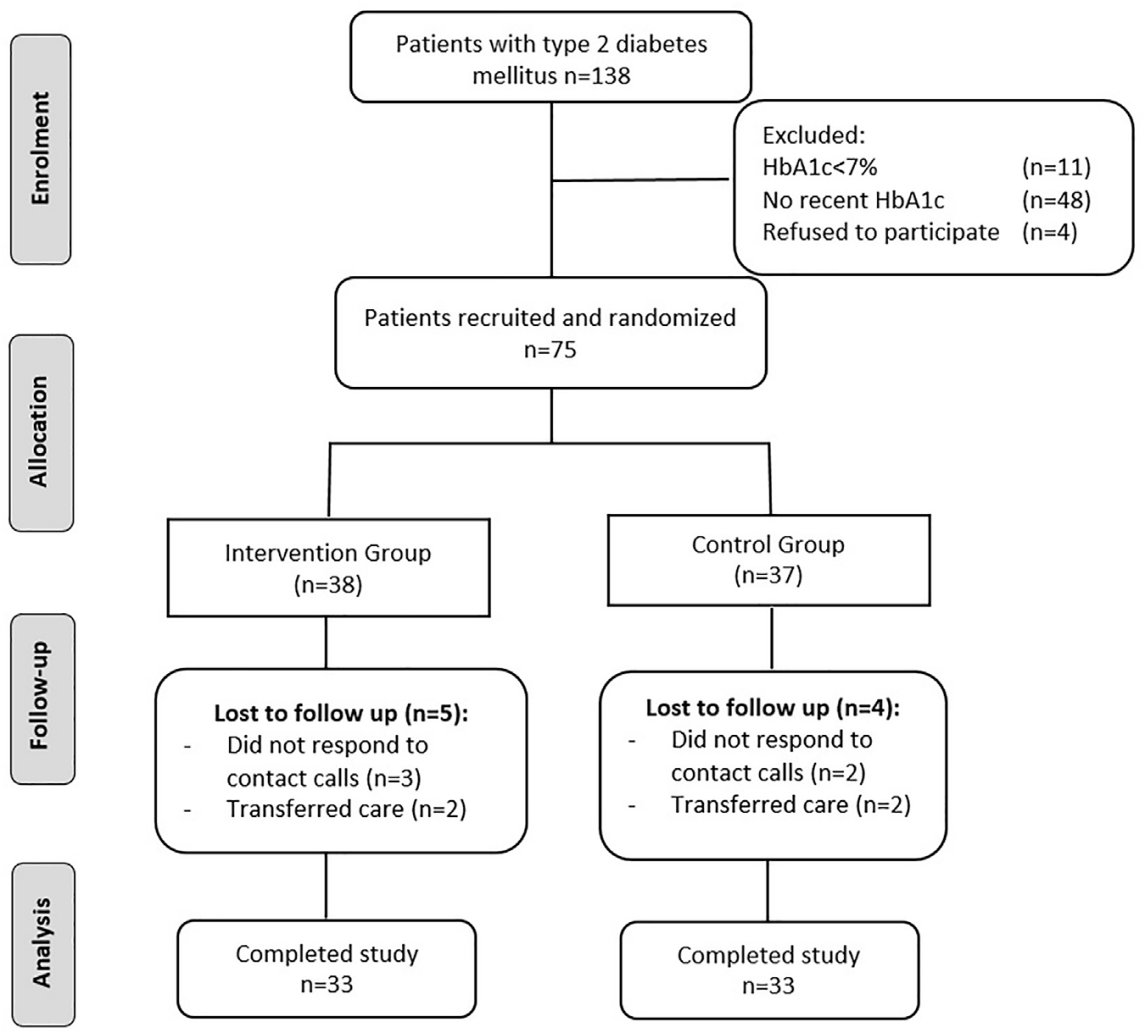
Study flow diagram in accordance with CONSORT guidelines (CONSORT—Consolidated Standards of Reporting Trials).

Of these seventy-five eligible participants, *n* = 38 were randomized to the pharmacist-led educational group and *n* = 37 were randomized to the usual care group. A total of sixty-six participants completed the 6-month study. The flow of study participants through the trial is presented in Figure 1 (compiled in accordance with the CONSORT guidelines). The baseline demographic characteristics of the intervention and control group are presented in Table 1. The demographic characteristics and clinical variables were similar in both groups at baseline (Table 1).

**TABLE 1:** Demographics of patients in the control and intervention groups (*N* = 66).

Parameter	Controls (*n* = 33)	Intervention (*n* = 33)	Total	*p*-values
Gender				
Male	16 (48.5)	20 (60.6)	36 (54.5)	.32^a^
Female	17 (51.5)	13 (39.4)	30 (45.5)	
Age (years) mean ± SD	51.72 ± 10.36	51.79 ± 12.8		
30–45 years	12 (36.4)	12 (36.4)	24 (36.4)	1.0^a^
>45–60 years	13 (39.4)	13 (39.4)	26 (39.4)	
> 60 years	8 (24.2)	8 (24.2)	16 (24.2)	
BMI (kg/m^2^)				
Normal (18.5 ≤ 25)	11 (33.3)	14 (42.4)	25 (37.9)	.41^a^
Overweight (25 ≤ 30)	10 (30.3)	12 (36.4)	22 (33.3)	
Obese (≥30)	12 (36.4)	7 (21.2)	19 (28.8)	
Smoking				
No	31 (93.9)	28 (84.8)	59 (89.4)	.43^b^
Yes	2 (6.1)	5 (15.2)	7 (10.6)	
Education				
No formal education	13 (39.4)	5 (15.2)	18 (27.3)	.28^b^
Primary level	4 (12.1)	5 (15.2)	9 (13.6)	
Secondary level	4 (12.1)	6 (18.2)	10 (15.2)	
High secondary level	4 (12.1)	7 (21.2)	11 (16.7)	
University level	8 (24.2)	10 (30.3)	18 (27.3)	
Family history of diabetes				
First degree relatives	21 (63.6)	24 (72.7)	45 (68.2)	.79^b^
Second degree relatives	1 (3.0)	1 (3.0)	2 (3.0)	
Both first and second deg. relatives	2 (6.1)	3 (9.1)	5 (7.6)	
No history	9 (27.3)	5 (15.2)	14 (21.2)	
Working status				
Jobless	3 (9.1)	1 (3.0)	4 (6.1)	.91^b^
Housewives/stay at home	14 (42.4)	13 (39.4)	27 (40.9)	
Business	3 (9.1)	4 (12.1)	7 (10.6)	
Doing Job	11 (33.3)	13 (39.4)	24 (36.4)	
Retired	2 (6.1)	2 (6.1)	4 (6.1)	
Diabetes duration (years)				
<5 years	10 (30.3)	13 (39.4)	23 (34.8)	.85^b^
5–9 years	11 (33.3)	8 (24.2)	19 (28.8)	
>9 ≤15 years	8 (24.2)	7 (21.2)	15 (22.7)	
≥15 years	4 (12.1)	5 (15.2)	9 (13.6)	
Anti-diabetic therapy				
Exclusively insulin	4 (12.1)	4 (12.1)	8 (12.1)	.65^b^
Combined with medication	17 (51.5)	13 (39.4)	30 (45.5)	
Oral Hypoglycemic agents only	12 (36.4)	16 (48.5)	28 (42.4)	
HbA1c value (%)	9.20 ± 1.22	9.00 ± 1.43		.22^c^

Data are *n* (%) or M ± SD. HbA1c, glycated hemoglobin; BMI, body mass index.

a Chi-square test.

b Fisher’s exact test.

c Independent-samples T-Test.

### Pharmacist-Led Intervention

Participants in the intervention group received two face-to-face self-care educational sessions by the pharmacist. A data collection form was designed to collect the data from the participants of the study regarding their demographic characteristics. However lab data were collected by the researchers from the patients’ lab profile. In the first session after collecting the baseline information about participants’ demographic characteristics, self-care practices and disease knowledge, the pharmacist educated the intervention participants (approximately 30–40 min) about diabetes, its symptoms, normal blood glucose levels and its monitoring, food choices for diabetes, importance of regular exercise and medicine use, and, diabetes-associated completions and their monitoring. In the second visit (12th week), pharmacist reinforced the intervention group participants about importance of adhering to diabetes-related self-care practices (approximately 15–30 min). During the second session, the participants were also inquired about barriers to self-care faced by them in the past 3-month, and remedial strategy or visit to physician was suggested, if required. The intervention group participants were supplemented with printed educational material and informatory brochures (in the Urdu language) about diabetes, diabetes-associated complications, and self-care activities. Besides these two face-to-face educational sessions, intervention group participants were also followed-up telephonically after every 4-week (till the end of trial).

American Diabetes Association (ADA) guidelines have been followed to design the contents of the educational intervention and informatory leaflets for people with diabetes. In order to make the educational intervention culturally-sensitive and patient-tailored, the contents of pharmacist-led educational intervention have been validated after two rounds of the Delphi-technique, involving six endocrinologists practicing in Pakistan. The detailed methodology of this trial and educational intervention is online available in this study’s published protocol (Bukhsh et al., 2018d). The pharmacist involved in delivering the educational intervention is registered with Punjab Pharmacy Council and was not involved in any healthcare delivery process to the study participants previously.

The participants in the control group received usual medical care, but were provided with the educational session and informatory brochures after the completion of this study.

### Outcomes

The primary outcome was change in glycated hemoglobin (HbA1c) level. Whereas, improvement in disease knowledge and diabetes-related self-care practices were the secondary outcomes of the study. Disease knowledge and self-care activities were examined by using Urdu versions of Diabetes Knowledge Questionnaire (DKQ) (Bukhsh et al., 2017a) and Diabetes Self-management Questionnaire (DSMQ) (Bukhsh et al., 2017b), respectively. Both of these study tools (DSMQ and DKQ) have been recently translated in to the Urdu language and psychometrically validated in type 2 diabetes patients in Pakistan. The scoring criteria of the DSMQ (Bukhsh et al., 2017b) and DKQ (Bukhsh et al., 2017a) have been described in detail in the published study protocol of this trial (Bukhsh et al., 2018d).

### Statistical Analysis

The data of the study were analyzed by using the Statistical Package for the Social Sciences (SPSS, Version 24 Inc., Chicago, IL, United States). Descriptive statistics, such numbers, standard deviation, and percentages were used to present and compare the baseline demographic characteristics of the control and experimental group participants. Chi-square test was used to test relationship between categorical variables of the participants in intervention and control. Independent-samples T-test (for continuous variables) and Fisher’s exact test (for categorical variables) was used to compare the group differences, whereas, Paired-Samples T-Test was applied to observe the difference between baseline and follow-up values. A *p*-value of less than .05 was considered significant for all analysis. The magnitude of effect of pharmacist-led intervention on primary outcome (HbA1c) was calculated by using Cohen’d (d = (M1−M2)/SD_*P*_). (Dunst et al., 2004).

## Results

### Clinical Outcomes

**FIGURE 2:**
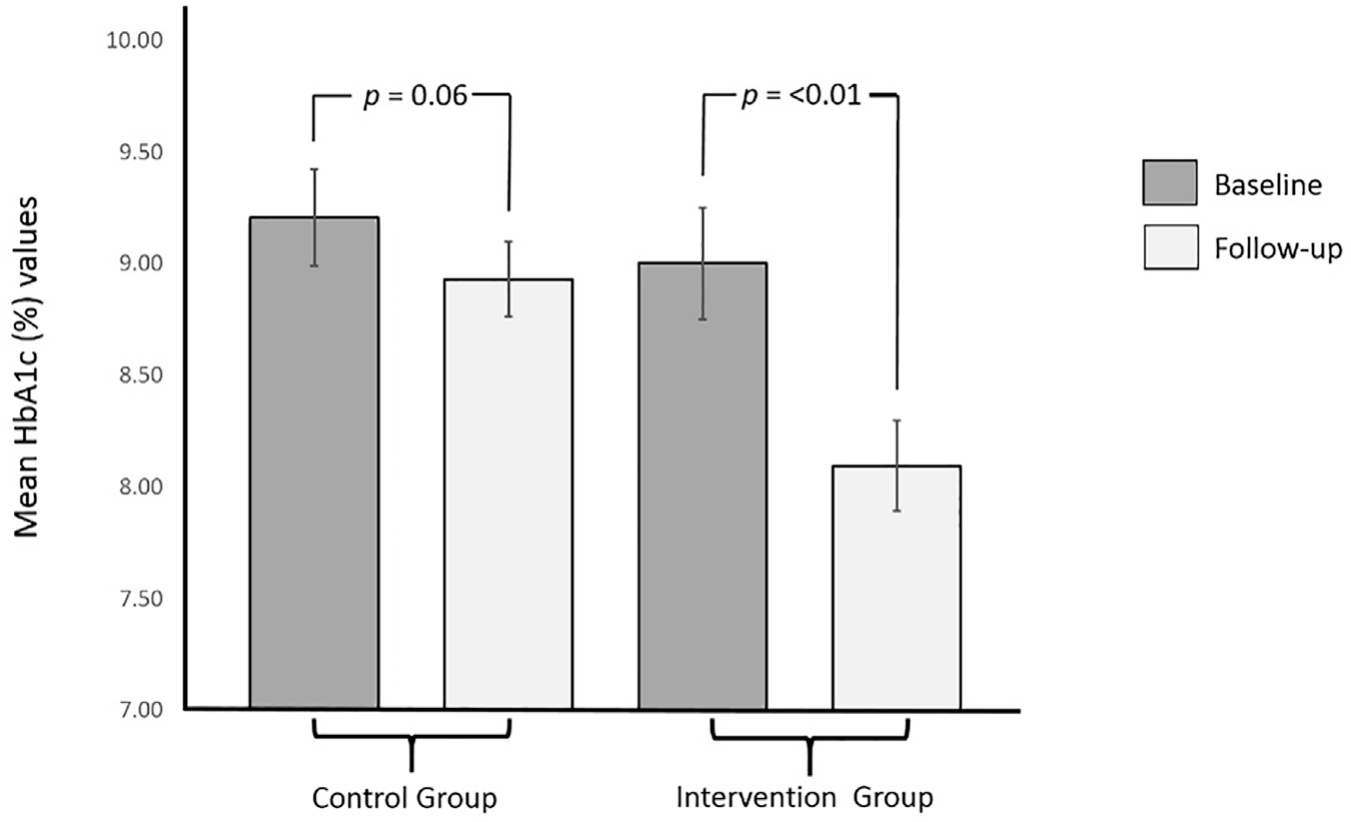
Mean baseline and final (after 6 months) HbA1c values in control and intervention groups.

**FIGURE 3:**
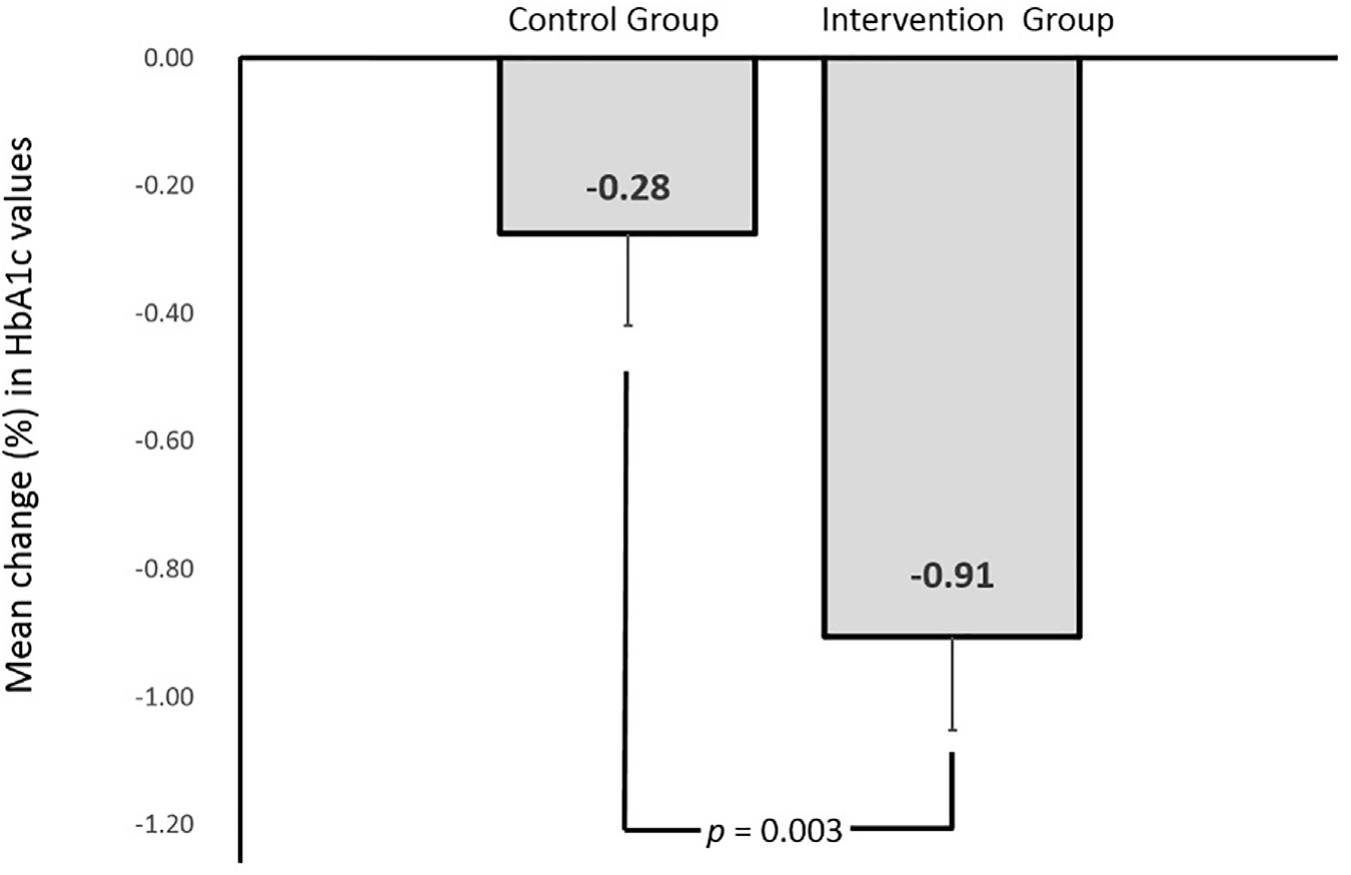
Mean change in HbA1c values of control and intervention groups after 6 months of study.

**FIGURE 4:**
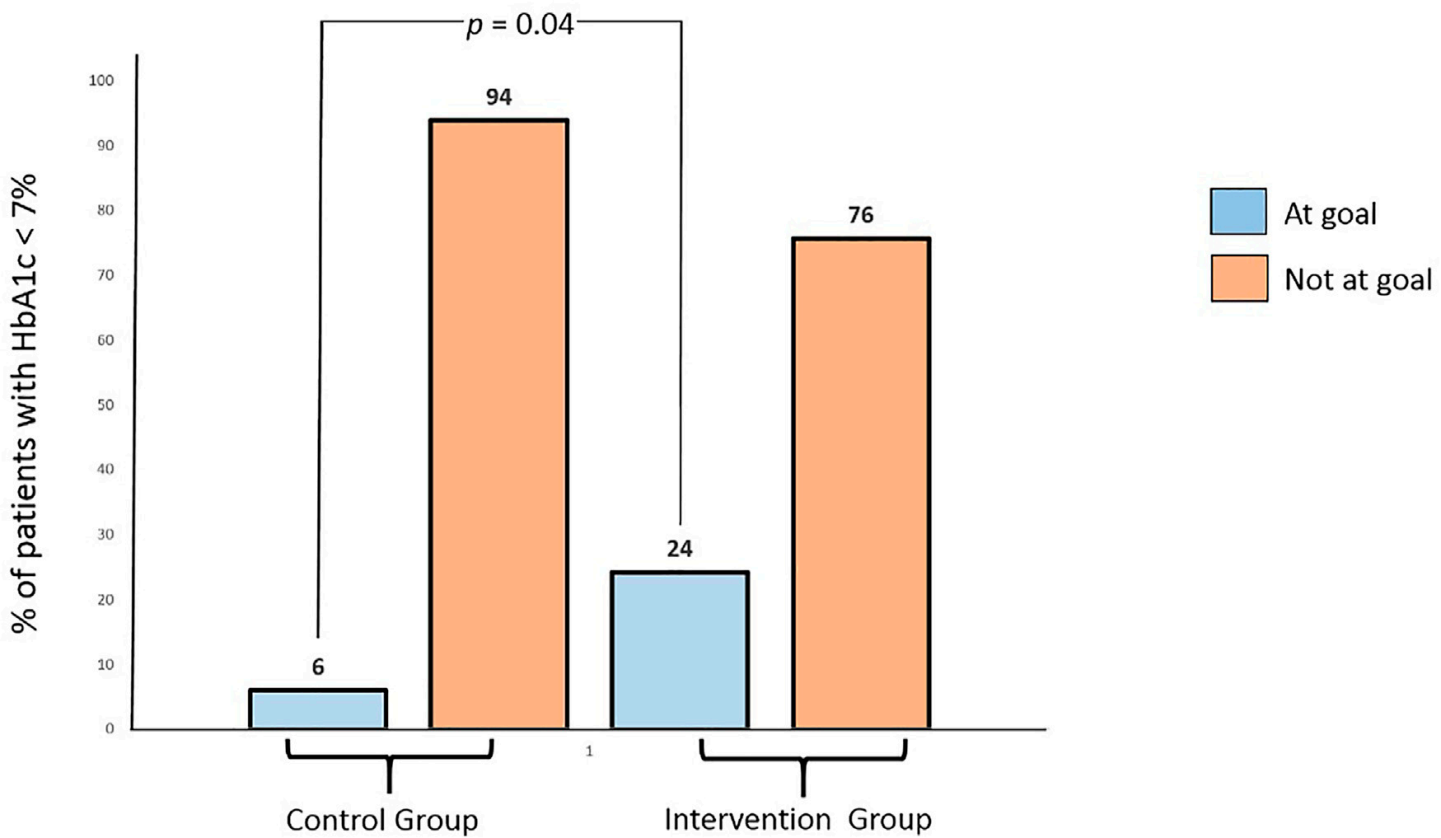
Percentage of patients in the control and intervention groups who achieved or did not achieve the goal of glycemic control (HbA1c < 7%) at the end of study (after 6 months).

Summary of changes in the outcomes of the study participants is presented in Table 2. Change in the levels of glycated hemoglobin (HbA1c) was the primary outcome of this trial. The baseline levels of glycated hemoglobin in the intervention (9.00 ± 1.43) and control (9.20 ± 1.22) group were similar at baseline (*p* > .05). Mean reducations for HbA1C level in the intervention group was significantly greater (.91%; *p* < .01) as compared to that in the control group (.28%; *p* = .06). Cohen’s d effect size of our intervention on glycated hemoglobin was .78. Figures 2, 3 shows the graphical representation of the changes in HbA1c levels of the control and the intervention group. After completion of the trial, the percentage of participants in this study who met the American Diabetes Association target of glycemic control (HbA1c < 7%) is presented in Figure 4.

**TABLE 2:** Comparison of participants’ scores in glycemic control and other health-related clinical outcomes in both groups at baseline and at 6 month’s follow up.

Outcome	Control group (*n* = 33)				Intervention group (*n* = 33)				Between groups mean difference
	Baseline	End of study	Mean difference	*p*-value^a^	Baseline	End of study	Mean difference	*p*-value^a^	*p*-value^b^
HbA1c	9.20 ± 1.24	8.93 ± .97	−.27	.06	9.00 ± 1.43	8.09 ± 1.16	−.91	<.01	<.01
Diabetes knowledge	12.00 ± 2.64	12.30 ± 2.42	.30	.11	12.79 ± 4.05	14.88 ± 3.57	2.09	<.01	<.01
DSMQ “Sum Scale”	5.92 ± 1.43	5.95 ± 1.39	.04	.31	5.36 ± 2.24	6.64 ± 1.58	1.28	<.01	<.01
Subscale “Glucose Management”	6.26 ± 1.88	6.48 ± 1.77	.22	.02	7.09 ± 2.06	8.20 ± 1.25	1.11	<.01	.001
Subscale “Dietary Control”	6.16 ± 1.63	6.11 ± 1.69	−.05	.32	4.85 ± 2.32	6.74 ± 1.74	1.89	<.01	<.01
Subscale “Physical Activity”	5.32 ± 1.75	5.35 ± 1.79	.03	.57	4.07 ± 2.81	5.35 ± 2.19	1.28	<.01	<.01
Subscale “Healthcare Use”	5.69 ± 1.49	5.59 ± 1.51	−.10	.37	4.44 ± 2.56	4.74 ± 2.41	.30	<.01	.01

All values are presented in Mean ± SD. HbA1c, Glycosylated hemoglobin; DSMQ, Diabetes Self-management Questionnaire.

a Paired-Samples T-Test.

b Independent-Samples T-Test.

### Self-Care Practices

A significant improvement (*p* < .05) in the self-care practices was observed in the intervention group in comparison to the usual care group. Scores for sum-scale and the four sub-scales of Diabetes Self-management Questionnaire (DSMQ) significantly (*p* < .05) improved in the participants of the intervention group. On the other hand, no significant improvement in DSMQ scores was noticed in the participants of the usual care group, except for Glucose Management (GM) sub-scale. Detailed result are presented in Table 2.

### Diabetes Knowledge

At the completion of trial, disease knowledge scores were significantly improved (*p* < .05) in the intervention group patients (*p* < .01) as compared to patients in the usual care group (Table 2).

## Discussion

This study is first of its kind to appraise the effect of pharmacist-led self-care education on glycemic control, self-care, and disease knowledge of type 2 diabetes patients in Pakistan having poor glycemic control. The findings of this study showed that reductions in glycated hemoglobin levels in the intervention group patients were significantly better than that of the usual care group. The outcomes of this study suggest that involving pharmacist in the provision of self-care education to people with diabetes may result in clinically and statistically significant improvements in blood sugar control, self-care practices, and diabetes knowledge.

Similar findings have been reported by Butt et al. (2016), in which a 6-month pharmacist-led educational program resulted in a mean reduction of 1.19% in the levels of HbA1c in the intervention group patients. The reductions in the mean glycated hemoglobin levels of the intervention group patients of this study are comparable to the findings of other published trials (Krass et al., 2007; Jahangard-Rafsanjani et al., 2015). Whereas, higher reductions in the levels of HbA1c have been reported, when pharmacist-led interventions were delivered for a duration longer than 6-month (Jameson and Baty, 2010; Ali et al., 2012; Chan et al., 2012; Jacobs et al., 2012; Chung et al., 2014).

In our study pharmacist educated the intervention group participants about diabetes, its associated complications, and importance of blood sugar control by improving self-care practices. Besides two interactive educational sessions, the pharmacist also kept a continuous monthly follow-up with the patients involved in this study via telephone, which resulted in improved self-care practices and better blood sugar control.

Glycemic control can significantly reduce the risk of diabetes-associated complications (Wishah et al., 2015). The patients with poor glycemic control (HbA1c ≥ 7%) are usually at high risk of acquiring diabetes-driven complications (Association AD, 2013). The UK prospective study (UKPDS) showed with every 1% decrease in HbA1c levels, there was 25% reduction in the rate of diabetes driven complications (Adler et al., 2000). Glycemic control depends on a variety of factors, such as disease knowledge and adherence to appropriate self-care practices. The target glycemic control by the American Diabetes Association (ADA) is HbA1c < 7% (Aschner, 2017). In our study, over 6 months, 25% of the intervention group participants achieved glycemic control, which was four times higher than the number of participant in the usual care group who achieved glycemic control. The percentage of intervention group patients who achieved glycemic control in our trial is comparable to the RCT conducted by Jarab et al. (2012), where, 23.4% of the patients achieved glycemic control.

The study findings indicated low levels of self-care practices in both groups at baseline, which could be attributed to poor knowledge about diabetes and its required self-care. The most frequently performed self-care activity in both groups was medication adherence, as indicated by high scores of “Glucose management” (sub-scale of DSMQ). On the other hand, exercise and diet were the least practiced self-care practices reported by the patients of both groups at the baseline. Poor self-care practices, especially for diet and exercise have also been reported in previous studies (Sarkar et al., 2006; Nelson et al., 2007; Wishah et al., 2015). These findings indicate people with diabetes have either inadequate knowledge about the importance of regular exercise and healthy eating or they feel it difficult to perform these self-care activities.

In our study education and counseling by the pharmacist, supplemented with informatory brochures (printed in the Urdu language) were the key features of pharmacist-led intervention. After 6-month of this pharmacist driven educational intervention led to a significant improvement in self-care practices and disease knowledge of the intervention group patients. Education and encouragement given by pharmacist helped the intervention group participants in improving their self-care practices and achieving the glycemic control.

## Strengths and Limitations

Currently, few educational programs running in diabetes clinics of Pakistan for people with diabetes, but the innovative approach involved in our study was, educating patients by the pharmacist about various aspects of diabetes-related self-care in addition of disease knowledge. One of the limitations of the study was its short duration to measure the sustainability of the desired clinical outcomes. Further studies with longer duration are required to examine these outcomes over a longer period, to maximize the reliability of the findings. Confounding factors such effect of nature of hypoglycemic agent, medication adherence, gender and age, have not been explored in this study, which requires to be explored in future. Another possible limitation to the study could be its single center design with relatively low sample size, which could limit the generalizability of findings over a large population. To produce more reliable and generalizable results, further multicenter randomized control trials are required.

## Conclusion

This study showed that pharmacist-led self-care education was linked with significant reductions in glycated hemoglobin levels of the intervention group participants as compared to the usual care group. Integrating the educational role of pharmacists in diabetes management may lead to a beneficial impact on the clinical outcomes of people with diabetes.

## Data Availability

The raw data supporting the conclusion of this article will be made available by the authors, without undue reservation.

## References

[B1] Adler A. I. Stratton I. M. Neil H. A. Yudkin J. S. Matthews D. R. Cull C. A. (2000). Association of Systolic Blood Pressure with Macrovascular and Microvascular Complications of Type 2 Diabetes (UKPDS 36): Prospective Observational Study. BMJ 321 (7258), 412–419. 10.1136/bmj.321.7258.412 10938049 PMC27455

[B2] Ali M. Schifano F. Robinson P. Phillips G. Doherty L. Melnick P. (2012). Impact of Community Pharmacy Diabetes Monitoring and Education Programme on Diabetes Management: A Randomized Controlled Study. Diabet Med. 29 (9), e326–33. 10.1111/j.1464-5491.2012.03725.x 22672148

[B3] Aschner P. (2017). New IDF Clinical Practice Recommendations for Managing Type 2 Diabetes in Primary Care. Diabetes Res. Clin. Pract. 132, 169–170. 10.1016/j.diabres.2017.09.002 28962686

[B4] Association AD (2013). Executive Summary: Standards of Medical Care in Diabetes--2013. Diabetes Care 36 (Suppl. 1), S4–S10. 10.2337/dc13-S004 23264424 PMC3537272

[B5] Association AD (2002). Standards of Medical Care for Patients with Diabetes Mellitus. Diabetes Care 25 (1), 213–229. 10.2337/diacare.25.1.213 11772918

[B6] Bukhsh A. Khan T. M. Lee S. W. H. Lee L. H. Chan K. G. Goh B. H. (2018). Efficacy of Pharmacist Based Diabetes Educational Interventions on Clinical Outcomes of Adults with Type 2 Diabetes Mellitus: A Network Meta-Analysis. Front. Pharmacol. 9, 339. 10.3389/fphar.2018.00339 29692730 PMC5902757

[B7] Bukhsh A. Khan T. M. Nawaz M. S. Ahmed H. S. Chan K. G. Lee L. H. (2018). Association of Diabetes-Related Self-Care Activities with Glycemic Control of Patients with Type 2 Diabetes in Pakistan. Patient Prefer Adherence 12, 2377–2385. 10.2147/PPA.S177314 30519003 PMC6235006

[B8] Bukhsh A. Khan T. M. Sarfraz Nawaz M. Sajjad Ahmed H. Chan K. G. Goh B. H. (2019). Association of Diabetes Knowledge with Glycemic Control and Self-Care Practices Among Pakistani People with Type 2 Diabetes Mellitus. Diabetes Metab. Syndr. Obes. 12, 1409–1417. 10.2147/DMSO.S209711 31616171 PMC6698595

[B9] Bukhsh A. Lee S. W. H. Pusparajah P. Khan A. H. Khan T. M. (2017). Psychometric Properties of the Urdu Version of Diabetes Knowledge Questionnaire. Front. Public Health 5, 139. 10.3389/fpubh.2017.00139 28702453 PMC5484766

[B10] Bukhsh A. Lee S. W. H. Pusparajah P. Schmitt A. Khan T. M. (2017). Psychometric Properties of the Diabetes Self-Management Questionnaire (DSMQ) in Urdu. Health Qual. Life Outcomes 15 (1), 200. 10.1186/s12955-017-0776-8 29025432 PMC5639758

[B11] Bukhsh A. Nawaz M. S. Ahmed H. S. Khan T. M. (2018). A Randomized Controlled Study to Evaluate the Effect of Pharmacist-Led Educational Intervention on Glycemic Control, Self-Care Activities and Disease Knowledge Among Type 2 Diabetes Patients: A Consort Compliant Study Protocol. Medicine (Baltimore) 97 (12), e9847. 10.1097/MD.0000000000009847 29561461 PMC5895327

[B12] Bukhsh A. Tan X. Y. Chan K. G. Lee L. H. Goh B. H. Khan T. M. (2018). Effectiveness of Pharmacist-Led Educational Interventions on Self-Care Activities and Glycemic Control of Type 2 Diabetes Patients: A Systematic Review and Meta-Analysis. Patient Prefer Adherence 12, 2457–2474. 10.2147/PPA.S180256 30538430 PMC6254657

[B13] Butt M. Mhd Ali A. Bakry M. M. Mustafa N. (2016). Impact of a Pharmacist Led Diabetes Mellitus Intervention on HbA1c, Medication Adherence and Quality of Life: A Randomised Controlled Study. Saudi Pharm. J. 24 (1), 40–48. 10.1016/j.jsps.2015.02.023 26903767 PMC4720029

[B14] Chan C. W. Siu S. C. Wong C. K. Lee V. W. (2012). A Pharmacist Care Program: Positive Impact on Cardiac Risk in Patients with Type 2 Diabetes. J. Cardiovasc. Pharmacol. Ther. 17 (1), 57–64. 10.1177/1074248410396216 21335480

[B15] Chen S. M. Creedy D. Lin H. S. Wollin J. (2012). Effects of Motivational Interviewing Intervention on Self-Management, Psychological and Glycemic Outcomes in Type 2 Diabetes: A Randomized Controlled Trial. Int. J. Nurs. Stud. 49 (6), 637–644. 10.1016/j.ijnurstu.2011.11.011 22209215

[B16] Chow S-C. Shao J. Wang H. Lokhnygina Y. (2017). Sample Size Calculations in Clinical Research. Chapman and Hall/CRC.

[B17] Chung W. W. Chua S. S. Lai P. S. Chan S. P. (2014). Effects of a Pharmaceutical Care Model on Medication Adherence and Glycemic Control of People with Type 2 Diabetes. Patient Prefer Adherence 8, 1185–1194. 10.2147/PPA.S66619 25214772 PMC4159395

[B18] Dunst C. J. Hamby D. W. Trivette C. M. (2004). Guidelines for Calculating Effect Sizes for Practice-Based Research Syntheses. Centerscope 3 (1), 1–10.

[B19] Hayward R. A. Krein S. L. Vijan S. (2005). Proactive Case Management of High-Risk Patients with Type 2 Diabetes Mellitus by a Clinical Pharmacist: A Randomized Controlled Trial. Am. J. Manag. Care 11, 253. 15839185

[B20] Hulley S. B. Cummings S. R. Browner W. S. Grady D. G. Newman T. B. (2013). Designing Clinical Research. Philadelphia: Lippincott Williams & Wilkins.

[B21] International Diabetes Federation (2017). IDF Diabetes Atalas. 10.1111/1753-0407.12644 29345068

[B22] Inzucchi S. E. Bergenstal R. M. Buse J. B. Diamant M. Ferrannini E. Nauck M. (2012). Management of Hyperglycemia in Type 2 Diabetes: A Patient-Centered Approach: Position Statement of the American Diabetes Association (ADA) and the European Association for the Study of Diabetes (EASD). Diabetes care 35 (6), 1364–1379. 10.2337/dc12-0413 22517736 PMC3357214

[B23] Jacobs M. Sherry P. S. Taylor L. M. Amato M. Tataronis G. R. Cushing G. (2012). Pharmacist Assisted Medication Program Enhancing the Regulation of Diabetes (PAMPERED) Study. J. Am. Pharm. Assoc. (2003) 52 (5), 613–621. 10.1331/JAPhA.2012.10183 23023841

[B24] Jahangard-Rafsanjani Z. Sarayani A. Nosrati M. Saadat N. Rashidian A. Hadjibabaie M. (2015). Effect of a Community Pharmacist-Delivered Diabetes Support Program for Patients Receiving Specialty Medical Care: A Randomized Controlled Trial. Diabetes Educ. 41 (1), 127–135. 10.1177/0145721714559132 25420946

[B25] Jameson J. P. Baty P. J. (2010). Pharmacist Collaborative Management of Poorly Controlled Diabetes Mellitus: A Randomized Controlled Trial. Am. J. Manag. Care 16 (4), 250–255. 20394460

[B26] Jarab A. S. Alqudah S. G. Mukattash T. L. Shattat G. Al-Qirim T. (2012). Randomized Controlled Trial of Clinical Pharmacy Management of Patients with Type 2 Diabetes in an Outpatient Diabetes Clinic in Jordan. J. Manag. Care Pharm. 18 (7), 516–526. 10.18553/jmcp.2012.18.7.516 22971205 PMC10437536

[B27] Krass I. Armour C. L. Mitchell B. Brillant M. Dienaar R. Hughes J. (2007). The Pharmacy Diabetes Care Program: Assessment of a Community Pharmacy Diabetes Service Model in Australia. Diabet Med. 24 (6), 677–683. 10.1111/j.1464-5491.2007.02143.x 17523968

[B28] Moreira R. C. Mantovani Mde. F. Soriano J. V. (2015). Nursing Case Management and Glycemic Control Among Brazilians with Type 2 Diabetes: Pragmatic Clinical Trial. Nurs. Res. 64 (4), 272–281. 10.1097/NNR.0000000000000104 26126062

[B29] Nelson K. M. McFarland L. Reiber G. (2007). Factors Influencing Disease Self-Management Among Veterans with Diabetes and Poor Glycemic Control. J. Gen. Intern. Med. 22 (4), 442–447. 10.1007/s11606-006-0053-8 17372790 PMC1829424

[B30] Sarkadi A. Rosenqvist U. (2004). Experience-Based Group Education in Type 2 Diabetes: A Randomised Controlled Trial. Patient Educ. Couns. 53 (3), 291–298. 10.1016/j.pec.2003.10.009 15186866

[B31] Sarkar U. Fisher L. Schillinger D. (2006). Is Self-Efficacy Associated with Diabetes Self-Management across Race/Ethnicity and Health Literacy. Diabetes care 29 (4), 823–829. 10.2337/diacare.29.04.06.dc05-1615 16567822

[B32] Van Eikenhorst L. Taxis K. van Dijk L. de Gier H. (2017). Pharmacist-led Self-Management Interventions to Improve Diabetes Outcomes. A Systematic Literature Review and Meta-Analysis. Front. Pharmacol. 8, 891. 10.3389/fphar.2017.00891 29311916 PMC5735079

[B33] Wishah R. A. Al-Khawaldeh O. A. Albsoul A. M. (2015). Impact of Pharmaceutical Care Interventions on Glycemic Control and Other Health-Related Clinical Outcomes in Patients with Type 2 Diabetes: Randomized Controlled Trial. Diabetes Metab. Syndr. 9 (4), 271–276. 10.1016/j.dsx.2014.09.001 25301007

[B34] Yaghoubi M. Mansell K. Vatanparastc H. Steeves M. Zeng W. Farag M. (2017). Effects of Pharmacy-Based Interventions on the Control and Management of Diabetes in Adults: A Systematic Review and Meta-Analysis. Can. J. Diabetes 41 (6), 628–641. 10.1016/j.jcjd.2017.09.014 29224636

